# A Computational Analysis of the Reaction of SO_2_ with Amino Acid Anions: Implications for Its Chemisorption in Biobased Ionic Liquids

**DOI:** 10.3390/molecules27113604

**Published:** 2022-06-03

**Authors:** Vanessa Piacentini, Andrea Le Donne, Stefano Russo, Enrico Bodo

**Affiliations:** 1 Chemistry Department, University of Rome “La Sapienza”, 00185 Rome, Italy; vanessa.piacentini@uniroma1.it (V.P.); stefano.russo@uniroma1.it (S.R.); 2 Department of Chemical, Pharmaceutical and Agricultural Sciences, University of Ferrara, 44121 Ferrara, Italy; andrea.ledonne@unife.it

**Keywords:** SO_2_ capture, amino acids, ionic liquids

## Abstract

We report a series of calculations to elucidate one possible mechanism of SO_2_ chemisorption in amino acid-based ionic liquids. Such systems have been successfully exploited as CO_2_ absorbents and, since SO_2_ is also a by-product of fossil fuels’ combustion, their ability in capturing SO_2_ has been assessed by recent experiments. This work is exclusively focused on evaluating the efficiency of the chemical trapping of SO_2_ by analyzing its reaction with the amino group of the amino acid. We have found that, overall, SO_2_ is less reactive than CO_2_, and that the specific amino acid side chain (either acid or basic) does not play a relevant role. We noticed that bimolecular absorption processes are quite unlikely to take place, a notable difference with CO_2_. The barriers along the reaction paths are found to be non-negligible, around 7–11 kcal/mol, and the thermodynamic of the reaction appears, from our models, unfavorable.

## 1. Introduction

SO_2_ is a colorless and poisonous gas and represents one of the main atmospheric pollutants generated by anthropic activities. Each year, around 150 million tons of SO_2_ are produced by various human activities, mainly burning fossil fuels, industrial processes, and energy production [1,2,3]. The anthropic SO_2_ is mainly produced by fossil fuel processing since SO_2_ is typically removed from liquid fuels before usage. Atmospheric SO_2_ can be oxidated to SO_3_, which reacts with water to form H_2_SO_4_, leading to the appearance of corrosive agents in the atmosphere. The ensuing soil and water acidification has been an environmental problem that has fortunately decreased its impact in recent years due to the adoption of measures aimed at a strong reduction of SO_2_ emissions, especially from gas exhaust due to the automotive compartment [4,5].

A reduction in SO_2_ pollution is achieved either by removing the gas from fuel prior to combustion or removing the gas after combustion by treatment of the flue gas (FDG, flue gas desulfurization) [6,7].

In FGD, different chemistries can be used to remove SO_2_, among others the most notable involve the use of limestone, magnesium oxide, and ammonia [8]. Most, if not all, of these absorption processes are irreversible and do not allow for SO_2_ recycling despite it being a useful chemical in industry. The only reversible absorption processes are those based on the use of amine solutions (in the same way they are employed for CO_2_ absorption [9]), but the process is far from being optimal due to the loss of amines in the stream gas.

Due to their low vapor pressure and their intrinsic ability to be functionalized for specific purposes [10], ionic liquids (ILs) have been proposed as possible green absorbing agents for CO_2_ and SO_2_ removal from gasses in industrial settings [8,11,12]. The absorption of SO_2_ in ILs can be physical [8,13,14] or chemical [15,16,17,18]. Our interest, in the present work, is the chemical absorption process whereby an SO_2_ molecule reacts with a specific group of the IL molecular ions and gives rise to a new chemical specie.

In analogy with what has been seen for CO_2_ [19,20], we explore the reaction mechanisms between an amino acid (AA) anion and SO_2_. Amino acid-based ILs (AAILS) have attracted the interest of a part of the research community because of their intrinsic biocompatibility [21,22,23,24,25,26,27,28,29] and, in particular, have been implemented as efficient chemisorbing media for CO_2_ absorption from flue gas [30,31,32,33,34,35,36].

A possible mechanism of the chemical reaction has already been proposed in [37], where the products have been characterized using FTIR spectroscopy. In [38], it has been shown how aqueous solutions of AAILs are efficient absorbent media and how SO_2_ can be regenerated after absorption by heating at 100 °C. In [39], a guanidium-based IL has been shown to undergo chemical reaction by means of its NH_2_ group to yield an –NH–SO_2_ structure.

This work is focused on understanding the mechanisms of the reaction between an AA anion and the SO_2_ molecule using ab initio calculations. We do not consider the problem of the diffusion of the SO_2_ molecule inside the fluid, nor do we include water in the calculation, but we shall assume an anhydrous IL and that the SO_2_ is already in the proximity of the reactive terminal of the AA anion, which is the NH_2_ group. To maintain generality, we had to adopt a simplified model that produces universal results, possibly independent of the many variables at play in real systems: we treat environmental effects using an approximate, continuum solvent model, and we do not include a cationic partner. While the latter sounds like an oversimplification, it is the only way to maintain the widest generality. While the cation does play a role in the reaction, it appears very difficult to assess it using ab initio modeling, since the binding motif and energy of an ionic couple in the bulk phase would be inevitably different from those that we can obtain in an isolated system. We also point out that the study of the effect of the cationic partner on the reaction mechanisms would require a different computational strategy and a systematic study of its presence upon varying coordinating properties, size, and steric shape. Such study lies outside the scope of the present work, which focuses exclusively on the reactivity of the anions.

Finally, to support our choice, we mention the data reported in [32,40] for the analogous reaction with CO_2_, that show how different cations have a limited effect in modifying both the reaction profile and the energetic barriers associated with the proton transfer step, especially when compared to the effect of a change in the AA anion.

## 2. Methods

The calculations have been performed using the g16 program [41] and the B3LYP functional combined with the 6−311+G(d,p) basis set, and corrected for dispersion interaction using the empirical GD3 approach of Grimme [42]. All structures have been fully optimized without constraints. Each stationary point has been further characterized by a harmonic frequency calculation, and the zero-point-energies (ZPE) have been added to the electronic energy. Thermodynamic functions such as the Gibbs free energy have been computed under room conditions. The uniqueness of the transition state has been verified using a suitable IRC (intrinsic reaction coordinate) calculation for all the structures presented. Electronic density and population analysis has been performed using Janpa [43].

All calculations have been performed in gas phase and in the solvent phase using the SMD, the universal solvation model based on solute electron density [44], with parameters of the acetonitrile solvent that can be used as representative of the dielectric screening acting in these liquids [19]. When possible, the SMD model has been modified to account for the measured dielectric constant of the IL.

**Scheme 1:**
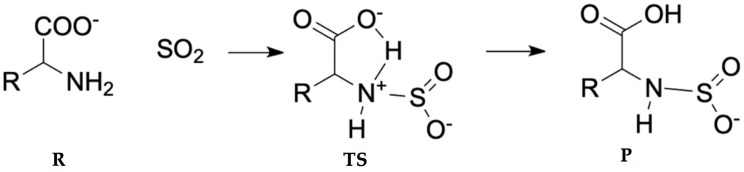
Generic reaction for the addition of SO_2_ to an AA anion.

The three AA anions that have been used to study the SO_2_ absorption process are: glycine (Gly), cysteine (Cys), and lysine (Lys), which have different chemical motifs on their side chains and could be representative of the variety of AA. The generic chemical reaction [37] between SO_2_ and the AA anion proceeds as reported in Scheme 1: Initially, there is a non-covalent complex, **R**, between the two molecules. This complex evolves through a transition state, **TS**, where a proton transfer occurs to produce the product, **P**. The specific mechanism depends on the type of AA anion and, in particular, in which way the proton transfer proceeds; for example, it can also take place between the –NH_2_^+^– and the SO_2_^−^ groups, thus producing, first, a sulfinic acid derivative, which undergoes an isomerization to the carboxylic acid form **P**. In addition, and as we shall see below, a second basic functional group of the AA can be, at least in principle, involved in the proton transfer step.

## 3. Results

### 3.1. The Glycinate Reaction

**Figure 1:**
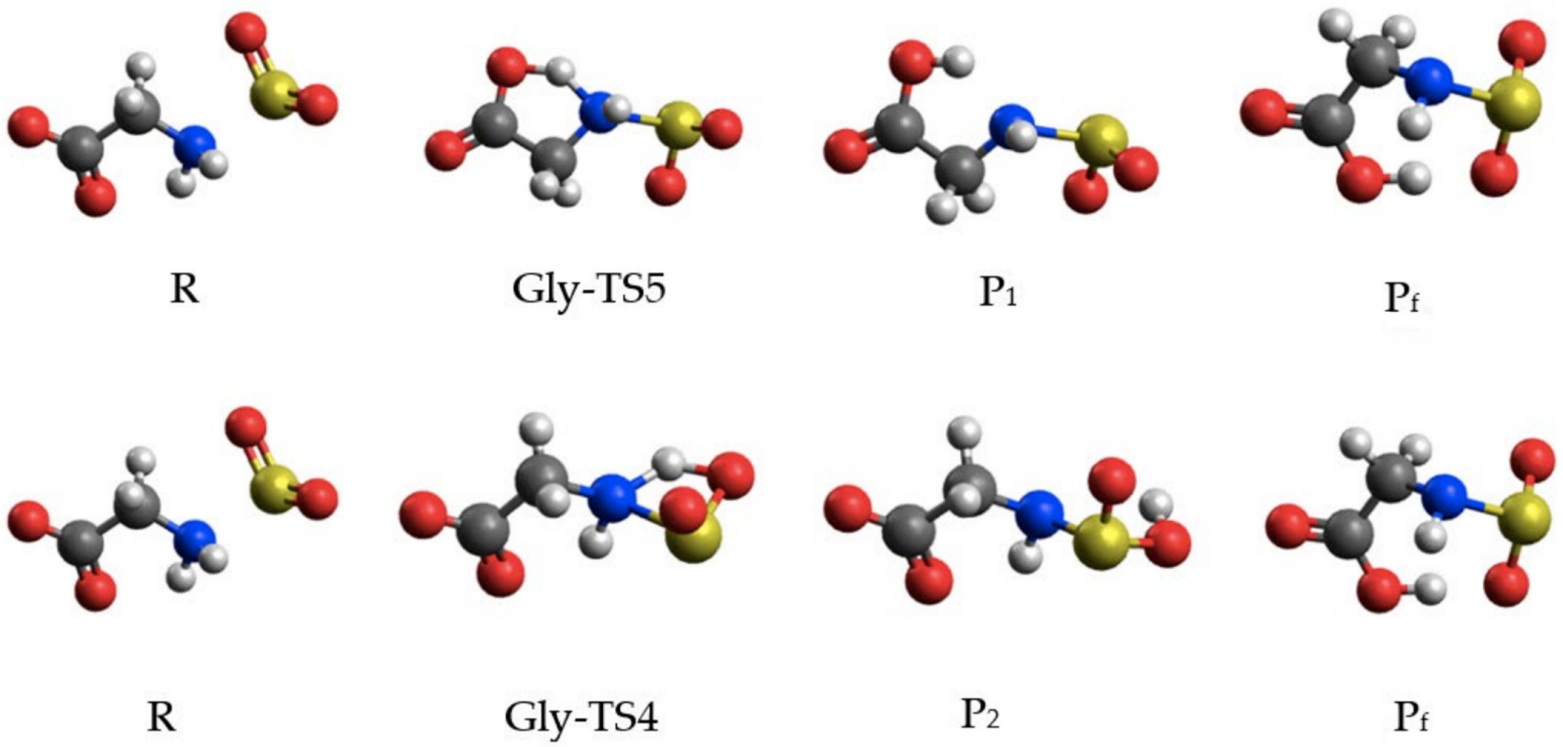
Stationary point geometries along the reaction of [Gly]^−^ with SO_2_. (**Top**): Path with a 5-mamber transition state that sees the transfer of a proton toward the carboxylate. (**Bottom**): Path involving a 4-member ring with proton transfer on the SO_2_. The last two structures in the top sequence are conformers, while those of the bottom sequence are isomers.

We begin our presentation by looking at the results on the simplest AA anion, i.e., Gly, that serves here as an exemplar case for all aliphatic AA anions. The optimized structures along two possible reaction paths (that differs from the nature of the proton transfer) are reported in Figure 1. The top one involves a transition state with a 5-member ring (**Gly-TS5**) structure, and the bottom one a 4-member ring (**Gly-TS4**). In the former, the proton transfer involves the carboxylate, and in the latter the SO_2_^−^ group. Both transition states require the formation of a positive charge formally located on the tetravalent nitrogen atom and of a negative charge on the oxygens of the –SO_2_ group.

While for a CO_2_ molecule the pre-reaction complex has a strong zwitterionic character, for SO_2_, the appearance of a charge separation takes place only when the process has already significantly evolved through the reaction coordinate [19]. Our calculations show that for SO_2_, there is almost no trace of zwitterion formation in the pre-reaction complex. This is already a clear sign of a diminished propensity of SO_2_ to react with the amino group.

The path involving **Gly-TS5** terminates to the product **P_1_** that has the proton on the carboxylate, as expected, and the final product **P_f_** is only a more stable conformer of the same structure. The path involving **Gly-PT4** produces a structure that has the proton on the –SO_2_ group that evolves to the same **P_f_** product of the first path by isomerization. The energetic path is in accordance with the expected acidity of the –SO_2_H and –CO_2_H groups.

**Figure 2:**
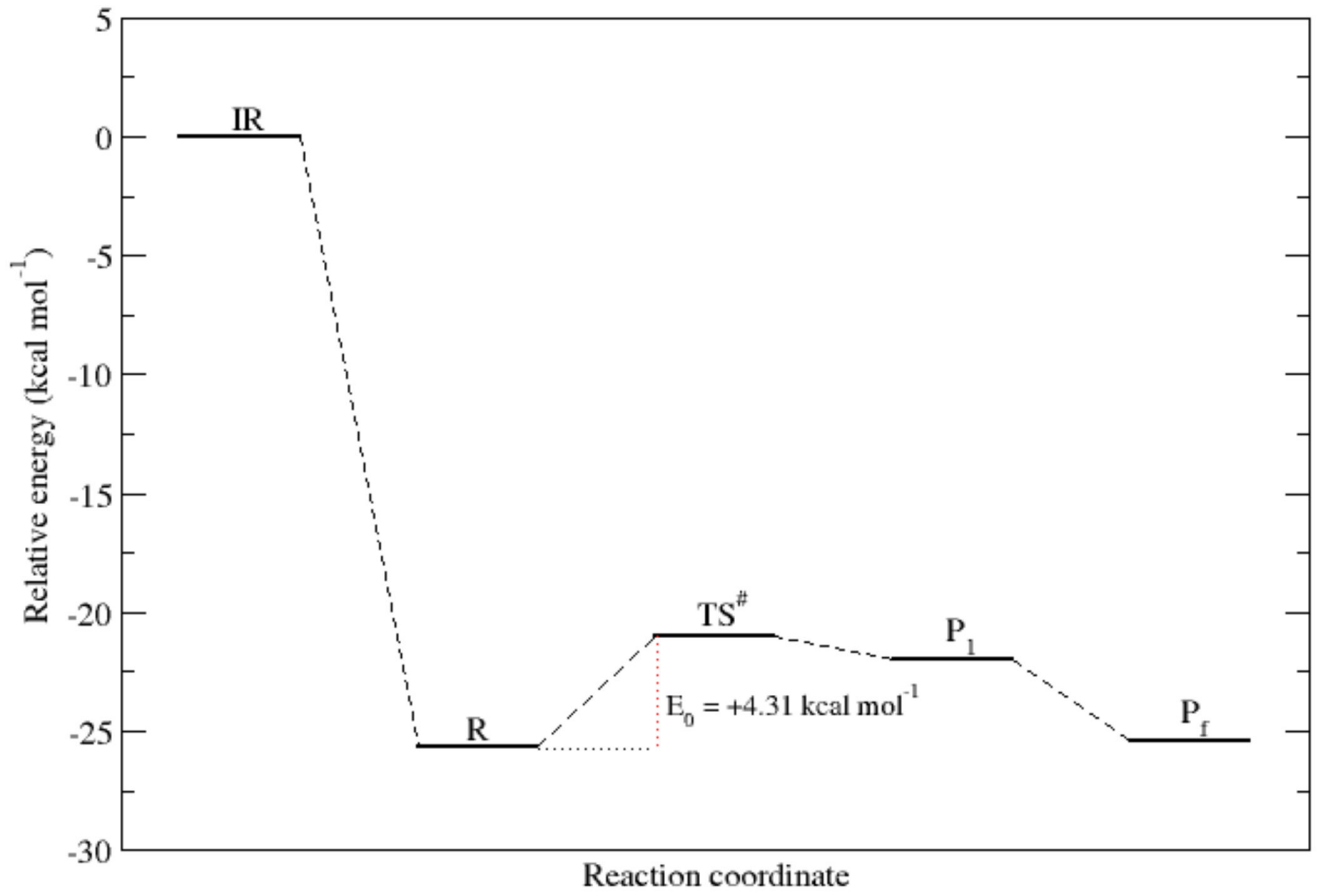
Reaction energies of the **Gly-TS5** path with respect to isolated reactants as computed using ZPE corrected electronic energies.

The energies of the **Gly-TS5** path in the gas phase are reported in Figure 2. With respect to the isolated reactants (**IR**), the reaction is highly exothermic with a $\Delta_{r}H$ of −26.4 kcal/mol and a $\Delta_{r}G$ of −13.0 kcal/mol. The reduced value of the reaction free energy is due to the unfavorable entropic contribution that emerges in an association reaction. If the initial reaction complex, **R**, is efficiently quenched by the environment, the addition reaction of SO_2_ to the AA anion is almost isoergonic, with little or no thermodynamic propensity.

**Figure 3:**
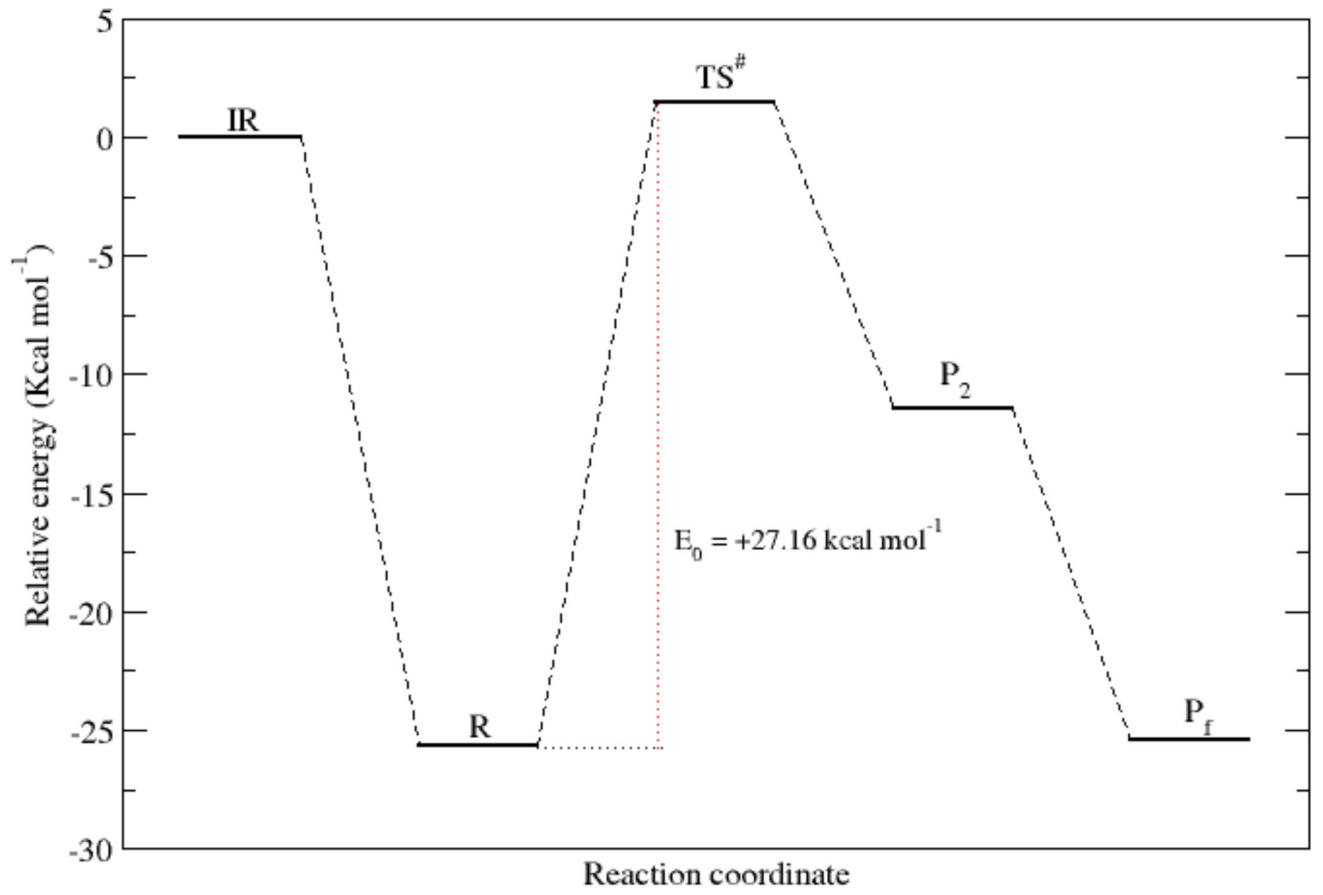
Reaction energies of the **Gly-TS4** path with respect to isolated reactants as computed using ZPE corrected electronic energies.

The energies of the **Gly-TS4** reaction path are reported in Figure 3. This path is hindered by a substantial activation barrier of about 27 kcal/mol that is due to the strain in the 4-member ring that characterizes the transition state. This path is obviously very inefficient, especially in an environment that can quench the initial reactant energy.

**Figure 4:**
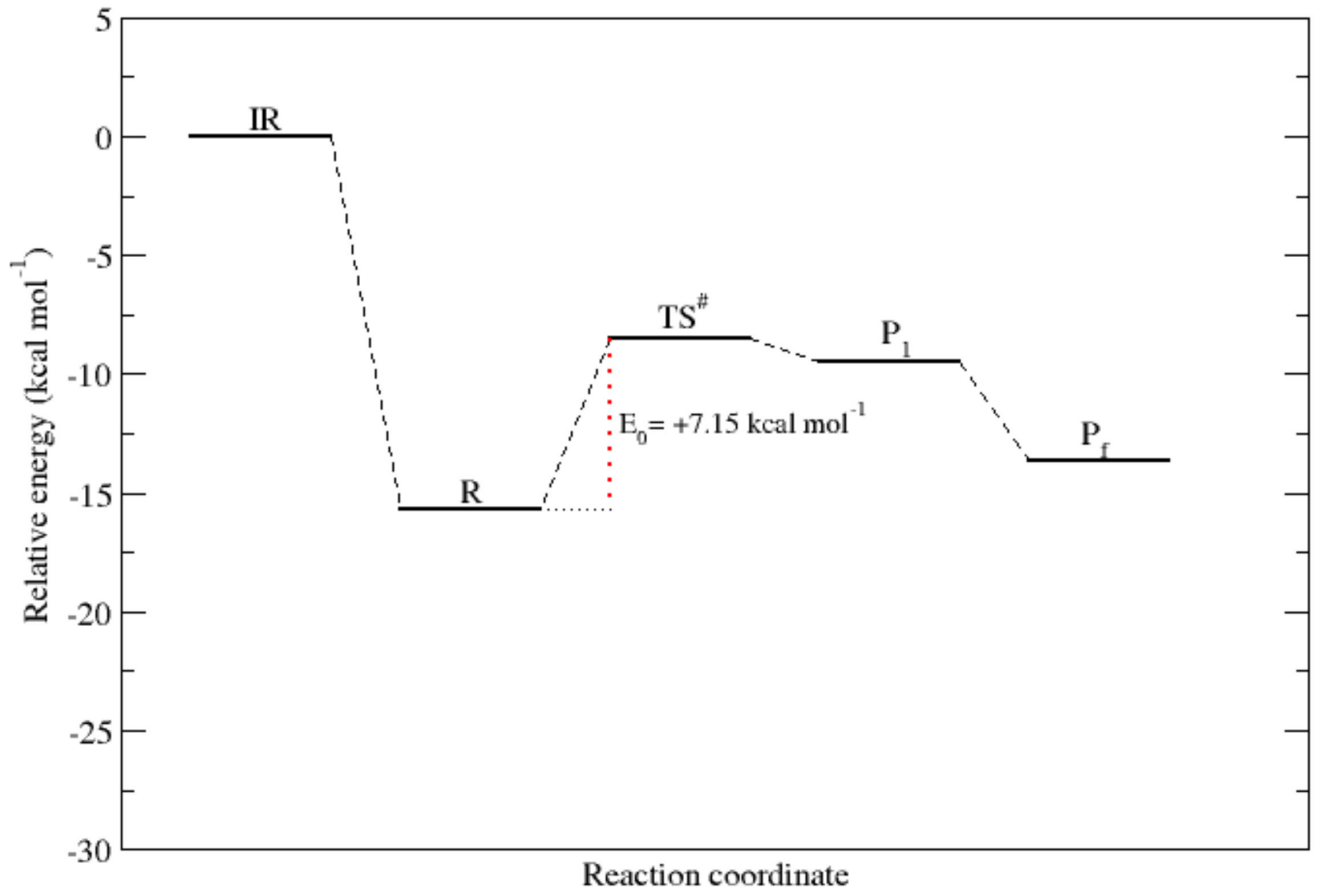
Reaction energies of the **Gly-TS5** path with respect to isolated reactants as computed using ZPE corrected electronic energies in the SMD solvent model.

The same calculations performed in a solvent model with a dielectric constant of 35.8 (acetonitrile) produced the numbers shown in Figure 4, where we report only the **Gly-TS5** path since the **Gly-TS4** path remains hindered by a barrier of 28.8 kcal/mol. The addition of the solvent has the effect of lowering the energy of the isolated reactants and to reduce the overall enthalpic gain to 14.82 kcal/mol and the corresponding $\Delta_{r}G$ to only −0.5 kcal/mol. The barrier with respect to the quenched reactants **R** is increased to 7.1 kcal/mol, a value that still allows the reaction to proceed quite efficiently at moderate temperatures.

Notably, the glycinate reaction with SO_2_ in a solvent medium becomes slightly endoergic if the pre-reaction complex **R** is sufficiently long-lived to be quenched by the environment. While this reduces the overall efficiency of the incorporation process, it also indicates that the above reaction can be reversible and that the AA anions can serve as a temporary storage of SO_2_.

**Figure 5:**
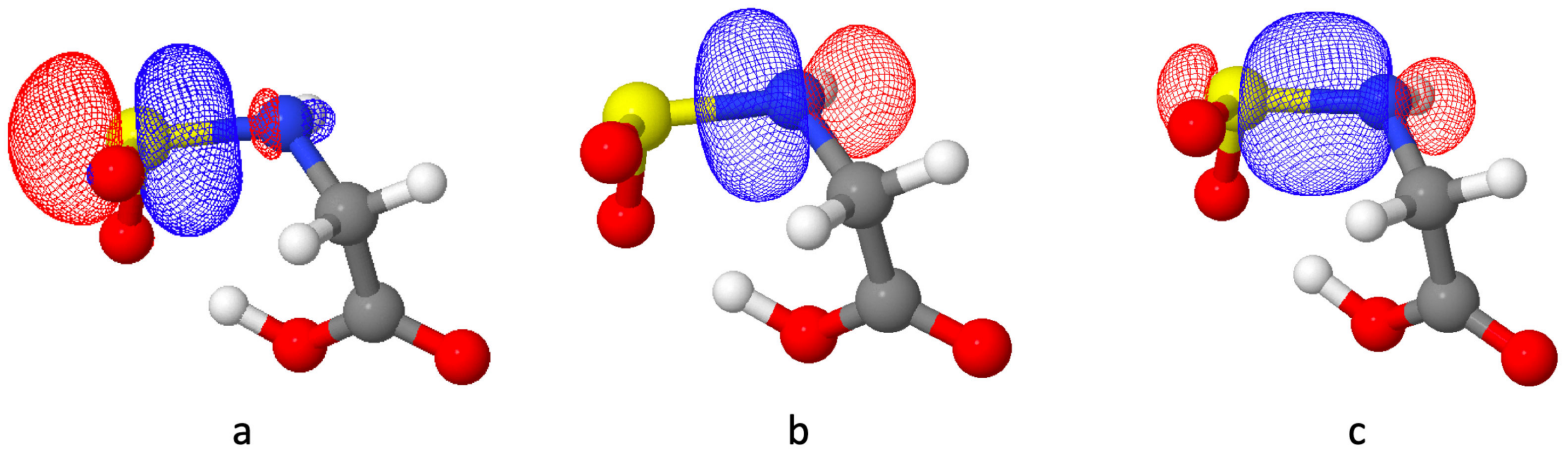
Localized molecular orbitals characterizing the N–S bond in the Gly derivative, **P_f_**. (**a**,**b**): Optimized atomic hybrids (LHOs) centered on S and N, respectively. (**c**): CLPO responsible for the bond, where the LHO centered on N accounts for 0.64% of the linear combination.

The final product is characterized by an unusual NH–SO_2_^−^ bond that is the sulfur equivalent of the N–C bond in carbamates. The N–S bond is significantly weaker than its N–C counterpart. Its bond distance (from our calculations) is large and is 1.8 Å for all three AA derivatives. The lowest energy dissociation path in vacuo is heterolytic and leads to negative –NH^−^ and neutral SO_2_. The adiabatic bond dissociation energies with respect to this fragmentation are 25.4, 22.2, and 24.0 kcal/mol for Gly, Cys, and Lys, respectively. This is a much lower bond energy when compared to that of the C–N bond in carbamates, which is about 100 kcal/mol [45]. In the sulfinic derivative, both the NH group and the SO_2_ are negatively charged with roughly half an electron each, as computed from natural orbital populations. The Wiberg–Mayer bond indices of the S–N bond for the three AA derivatives are 0.85, 0.72, and 0.70 for Gly, Cys, and Lys, respectively, again pointing to a weak single bond. The N–S bond can be described by the localized molecular orbital (CLPO) arising from the combination of two optimally paired hybrids [43], where the contribution of the N hybrid is the dominant one, as shown in Figure 5 for the Gly derivative.

### 3.2. The Cysteinate Reaction

**Figure 6:**
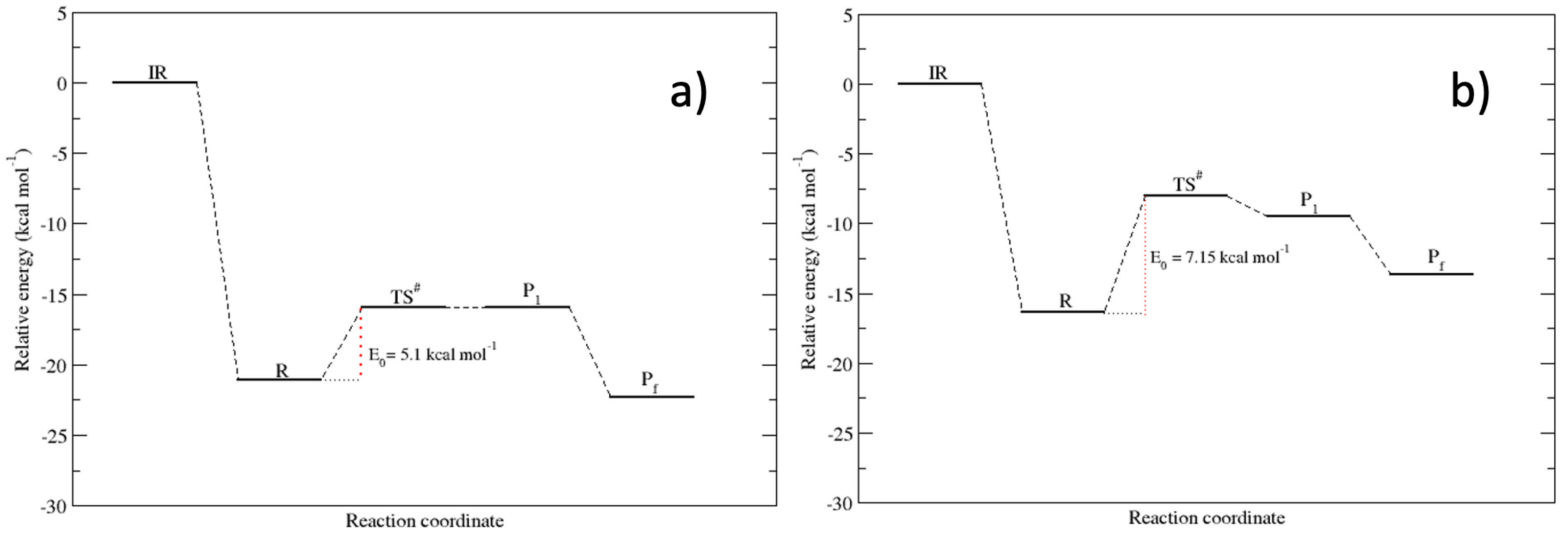

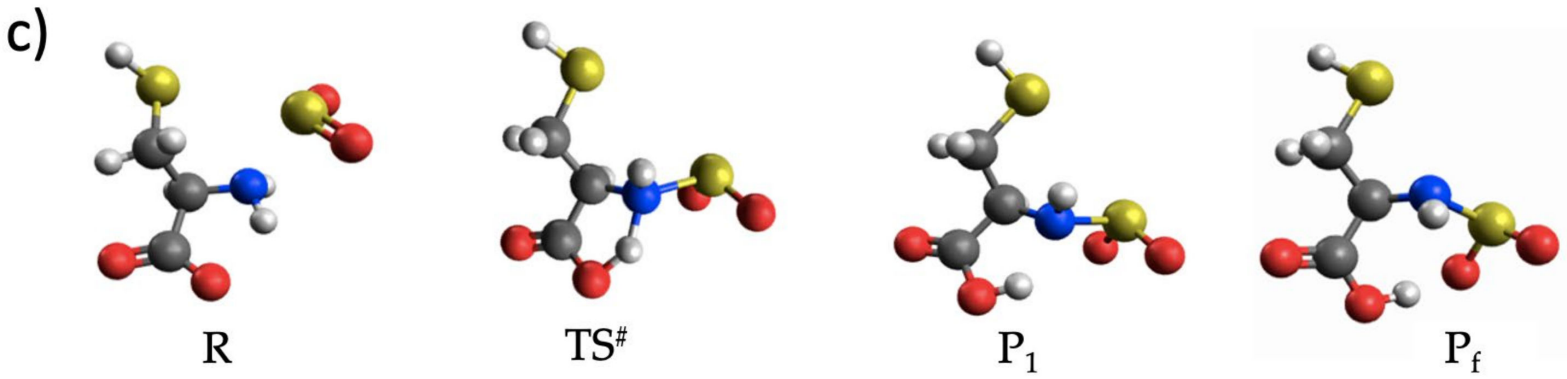
Reaction energies of the **Cys-TS5** path with respect to isolated reactants as computed using ZPE corrected electronic energies. (**a**): Reaction in the gas phase. (**b**): Reaction in the SMD solvent model. (**c**): Optimized geometries in the gas phase of the stationary points along the reaction path.

The reaction with a cysteinate anion proceeds substantially in the same way. A first possible path (**Cys-TS5**) for the reaction with SO_2_ requires a 5-member transition state and a proton migration toward the carboxylate group. This path in the gas phase, as in the Gly case, has a low activation barrier of 5.1 kcal/mol, and the $\Delta_{r}H\;\mathrm{and}\;\Delta_{r}G$ are −23.19 and −9.65 kcal/mol, respectively. The same calculation repeated in the SMD model, with the dielectric constant of 9.5 (which is the measured dielectric constant for the [Ch][Cys] IL reported in [46]), yields a $\Delta_{r}H$ of −14.4 kcal/mol and a $\Delta_{r}G$ of −1.0 kcal/mol. The reaction paths in vacuo and in solvent are reported in Figure 6, along with the geometries of the stationary points.

As we have shown in [47], the cysteinate anion can present itself in an isomeric form, where the proton of the thiol group has migrated onto the carboxylate. The result is that this isomeric form of the AA anion can bind the SO_2_ molecule only via the reaction path that requires a proton transfer from the –NH_2_ group through the highly unstable transition state characterized by a 4-member ring. Needless to say, this path is hindered by a large activation barrier of 27.1 kcal/mol. In addition, the overall reaction free energy becomes positive and equal to 5.8 kcal/mol, thus rendering this reaction channel extremely unlikely to be of any relevance for the pollutant absorption.

We tried to locate a possible reaction path that involved a proton transfer toward the thiolate group, but none of our attempts were successful. In other words, it appears that the –SH group does not play an active role in the SO_2_ absorption processes.

### 3.3. The Lysinate Reaction

A third set of calculations were repeated on the Lys anion, where it can be argued that the additional basic NH_2_ group on the side chain could play a role in the reaction mechanism or even give rise to a bimolecular absorption path, in which both amino groups are converted to sulfinic acids.

**Figure 7:**
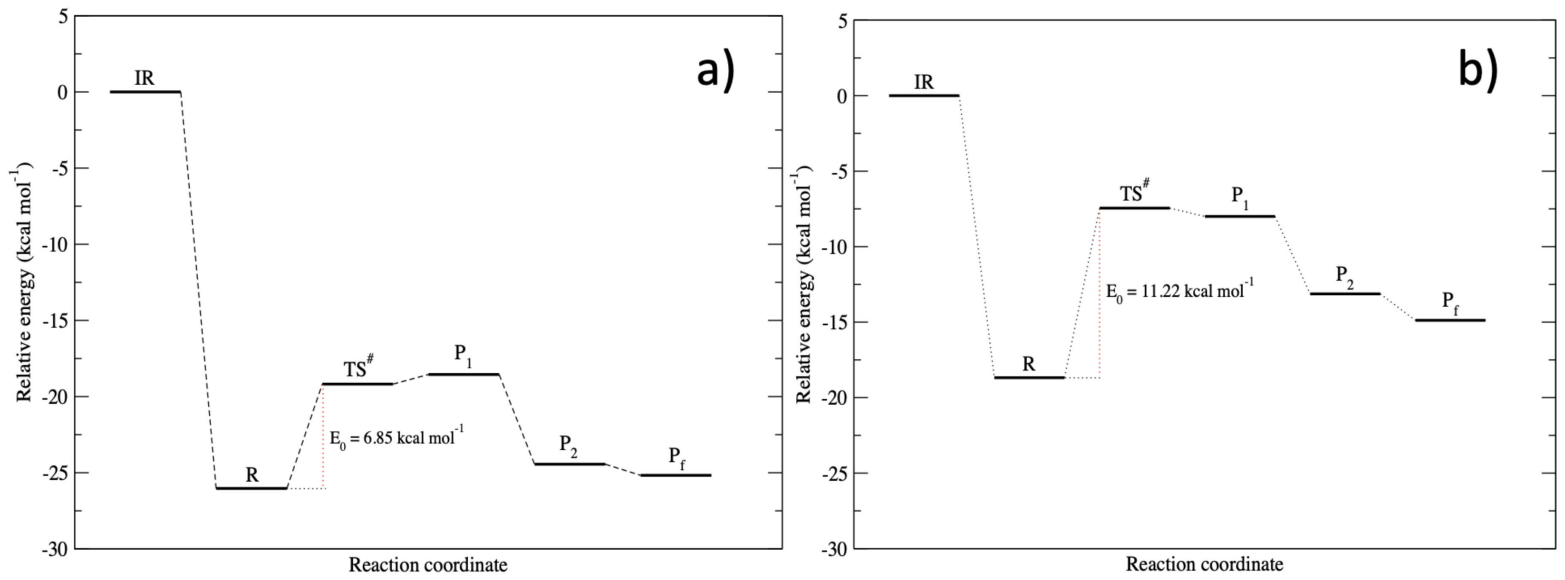

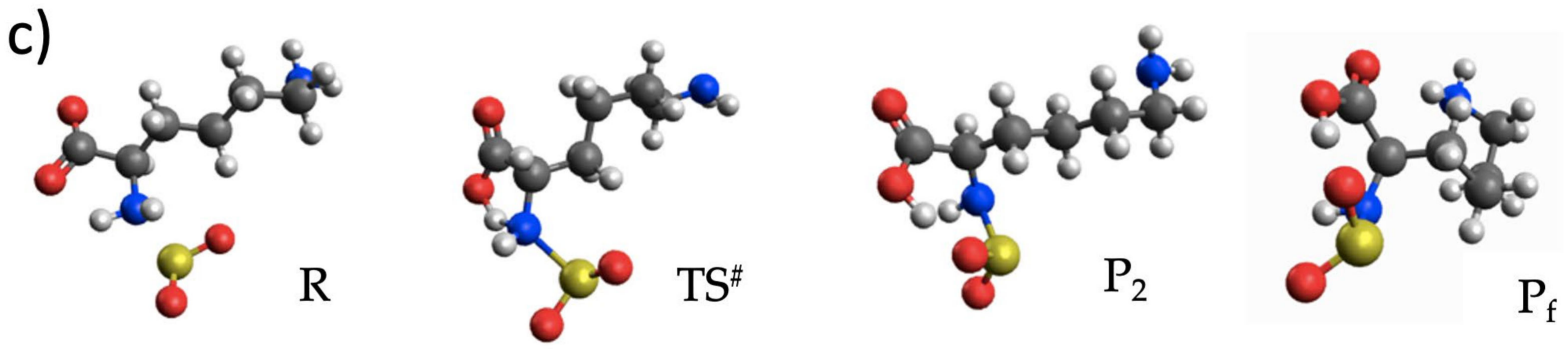
Reaction energies of the **Lys-TS5** path with respect to isolated reactants as computed using zero-point energy-corrected electronic energies. (**a**): Reaction in the gas phase. (**b**): Reaction in the SMD solvent model. (**c**): Optimized geometries in the gas phase of the stationary points along the reaction path. The **P_1_** product is a high-energy isomer of **P_2_**.

The analogous of the **Gly-TS5** and **Cys-TS5** mechanisms is also active for Lys and is reported in terms of energies and structures in Figure 7. The barriers in the gas phase and in the model solvent (with a dielectric constant of 9.8 [46]) are 6.8 and 11.2 kcal/mol, respectively. For the gas phase, the $\Delta_{r}H\;\mathrm{and}\;\Delta_{r}G$ are −26.4 and −11.8 kcal/mol, respectively. These values are reduced to −15.6 and −2.4 kcal/mol in the solvent model.

We have tried to trace a possible reactive path that involved the side chain NH_2_, but this group seems impervious to the SO_2_ attack. This is probably due to its higher basicity and to the absence of the carboxylate in β that can activate the reaction. This result, albeit negative, is important because the SO_2_ molecule appears less reactive than CO_2_, especially toward the AA side chains. It therefore appears unlikely that a single AA anion would be able to capture more than one SO_2_ molecule unless its molecular structure is purposedly engineered by placing more than one “activated” amino group. In comparison, we defer the reader to our study in [20], where we had shown how efficient a bimolecular reaction of the same AA can be when CO_2_ is involved.

## 4. Conclusions

This study attempted to elucidate a possible reaction mechanism based on the chemisorption of SO_2_ in biobased ionic liquids, namely, its reaction with the amino group of the AA anion. The model employed here did not account for the problem of SO_2_ diffusion in the liquid, and instead only focused on the reactive step once the SO_2_ had the chance to attack the amino group of the AA anion. Solvation and stabilization due to a dielectric has been nevertheless considered by using an approximate continuum solvent model. This approach, albeit quite simplified, has already been successfully used by us to illustrate the mechanisms at the basis of the quite efficient absorption of CO_2_ in these liquids [19,20].

In comparison to CO_2_, where a set of reaction mechanisms were found to be essentially barrierless and strongly exoergic with negative $\Delta_{r}G$, SO_2_ was less reactive towards the AA anions. This reduced reactivity substantially prevents SO_2_ from taking advantage of the presence of specific functional side chains, and the only mechanism that is viable is a direct addition of it to the amino group next to the carboxylate, a reactive path that should be common to all AA anions.

The mechanism involves passing through a cyclic transition state, where one of the protons initially belonging to NH_2_ is transferred to the carboxylate, neutralizing it. The resulting deprotonated sulfinic acid derivative is the final product. We have tried to find other mechanisms that involve the side chain, but we were unable to find any other efficient route. In contrast with the case of CO_2_, where the slightness or even absence of kinetic barriers makes the reaction extremely efficient, SO_2_ appears less prone to chemisorption due to the appearance of non-negligible barriers and to a much less exoergic thermodynamic of the involved reactions. In general, the barrier to the chemisorption reaction appeared to lie in the 7–11 kcal/mol interval, while the reaction free energies were only slightly negative, with values between −0.5 and −2 kcal/mol. The reason for this behavior lies in the relative weakness of the N–S bond when compared to its analogous in carbamates (the result of the CO_2_ reaction). The bond tends to break heterolytically, leading to neutral SO_2_, and its dissociation requires only 22–25 kcal/mol. This last aspect, albeit reducing the overall reaction rate, did however indicate a facile reversibility of the absorption reaction, thus pointing to a possible use of these materials as temporary storage for SO_2_.

While in the case of CO_2_ the overall absorption process, due to the high rate of the absorption reaction, seemed to be dominated by the rate of diffusion of the molecule in the liquid, for SO_2_, the chemical step is very likely to be much slower (especially at room temperature) and thermodynamically inefficient. This means that the absorption of SO_2_ in these liquids can easily be dominated by physisorption, with chemisorption being only a minor factor contributing to their overall intake capacity.

## Data Availability

The data presented in this study are available upon reasonable request from the corresponding author.
